# Recent Advances on Rapid Detection Methods of Steroid Hormones in Animal Origin Foods

**DOI:** 10.3390/bios15040216

**Published:** 2025-03-27

**Authors:** Yaohui Xue, Jinhua Li, Ming Ma, Pan Fu, Sihua Qian, Chao Han, Yuhui Wang

**Affiliations:** 1 Ningbo Institute of Materials Technology and Engineering, Chinese Academy of Sciences, Ningbo 315201, China; xueyaohui@nimte.ac.cn; 2 School of Chemical Engineering, North China University of Science and Technology, Tangshan 063210, China; fupan@nimte.ac.cn (P.F.); qiansihua@nimte.ac.cn (S.Q.); 3 Ningbo Customs Technology Center, Ningbo 315048, China; 13736012141@163.com (J.L.); 15867572519@163.com (M.M.)

**Keywords:** food safety, steroid hormones, rapid test, electrochemical, colorimetric, immunochromatographic, surface-enhanced Raman spectroscopy, fluorescence detection

## Abstract

Animal-derived foods constitute a crucial source of nutrients for humans. The judicious application of steroid hormones in the breeding process can serve multiple purposes, including growth promotion, weight gain, and anti-inflammatory effects, among others. However, excessive misuse poses a considerable risk to both food safety and consumer health. Currently, the primary means of detecting steroid hormones involve liquid chromatography, gas chromatography, and their combination with mass spectrometry. These methods necessitate advanced instrumentation, intricate pretreatment procedures, and the expertise of specialized laboratories and technicians. In recent years, the swift evolution of analytical science, technology, and instrumentation has given rise to various rapid detection techniques for steroid hormone residues, providing a robust technical foundation for ensuring food safety. This review commences by delineating the roles of steroid hormones, the associated residue hazards, and the pertinent residue restriction standards. Subsequently, it delves deeply into the analysis of the most recent rapid detection techniques for steroid hormones, ultimately culminating in an assessment of the challenges currently confronting the field, along with an exploration of potential future advancements. We sincerely hope that this review will inspire and provide valuable insights to the pertinent researchers.

## 1. Introduction

In the breeding of food animals, steroid hormones are employed for a variety of purposes, including artificial reproduction, sex transformation, and the treatment of inflammatory conditions and disease resistance [1,2,3]. These hormones are also used in veterinary drugs used in farming such as hormones and antibiotics, which are categorized as endocrine-disrupting chemicals (EDCs) [4,5]. Since the 1970s and 1980s, there have been numerous reports of endocrine disruption associated with anabolic steroid residues in food. It has been demonstrated that even at residual levels that are very low, these hormones can still cause long-term harm [6,7]. They cannot be removed from water bodies by conventional wastewater treatment systems, and they remain in the environment, causing long-term environmental impacts [8,9].

Over the past decades, researchers have developed several methods to detect steroid hormone residues in various matrices, including gas chromatography (GC), liquid chromatography (LC), gas chromatography–mass spectrometry (GC–MS), liquid chromatography–mass spectrometry (LC–MS) techniques, enzyme-linked immunosorbent assay (ELISA), and others [10]. The most commonly employed detection technique is high-performance liquid chromatography (HPLC), which exhibits high sensitivity [11]. However, due to the polarity and low volatility of steroids, the analysis of small molecules such as steroids typically necessitates pre-column derivatization [12,13]. These pre-treatments are frequently complex and time-consuming, requiring the utilization of substantial quantities of organic solvent. Incomplete derivatization can also result in low recoveries and inaccurate results [14,15]. This necessitates a high level of professionalism from the analytical operator. Furthermore, the instrument itself is expensive, large, and requires a professional working environment, and thus these methods are not suitable for on-site testing.

**Figure 1:**
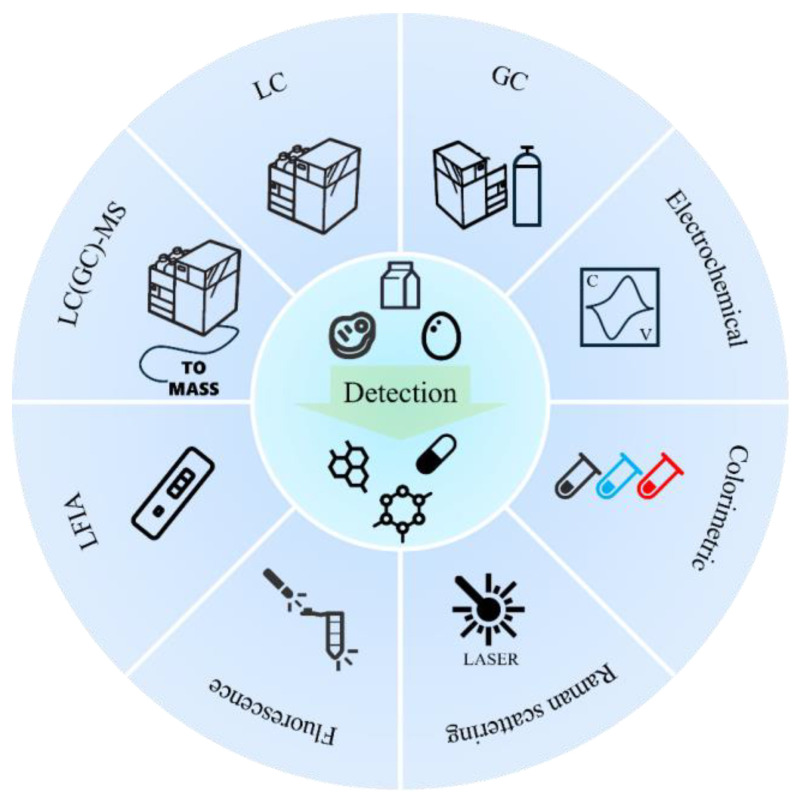
Common detection techniques and methods for steroid hormones in foods.

In recent years, there has been a diversification of the food industry, which has led to a shift in food testing methods towards greater speed, sensitivity, portability, simplicity, and low-cost high-throughput operations. Figure 1 illustrates the main methods currently used for the detection of steroid hormones in food. Diverse quick detection methods based on electrochemistry [16], colorimetry [17,18], fluorescence/phosphorescence [19], Raman spectroscopy [20,21], and lateral flow immunoassays (LFIA) [22,23,24] have been developed for rapid screening of steroid hormones in food. Compared to traditional LC–MS and GC–MS methods, these techniques require less or no sample preparation, small portable instruments, or no instruments at all, thereby enhancing assay efficiency and substantially decreasing assay costs [25]. However, to the best of our knowledge, there is no review associated with quick detection methods for steroid hormones. In this review, we provide an overview of the development and application of rapid methods for the determination of steroid hormones over the past decades. It outlines the advantages and limitations of these methods, with the aim of providing a reference for the rapid detection of steroid hormones in food safety and promoting the development and application of new detection methods in the future.

## 2. Rapid Tests for Steroid Hormones

### 2.1. Electrochemical Methods

Electrochemical (EC) techniques offer a number of advantages in the detection of steroid hormones, including low cost, high sensitivity, fast response, easy operation, and real-time monitoring [26]. Among the various electrochemical detection techniques, voltammetry is the most commonly used method, as it allows for the quick measurement of redox potential during the process [27,28]. This method is considered accurate, fast, and highly sensitive. Within the field of voltammetry, different techniques are employed for the qualitative and quantitative detection of substances. These include cyclic voltammetry (CV), square wave voltammetry (SWV), linear scanning voltammetry (LSV), and differential pulse voltammetry (DPV). All these methods are designed on the adoption of a specific voltage to the working electrode, followed by the detecting of any changes in current over the range of the applied voltage [29,30].

**Figure 2:**
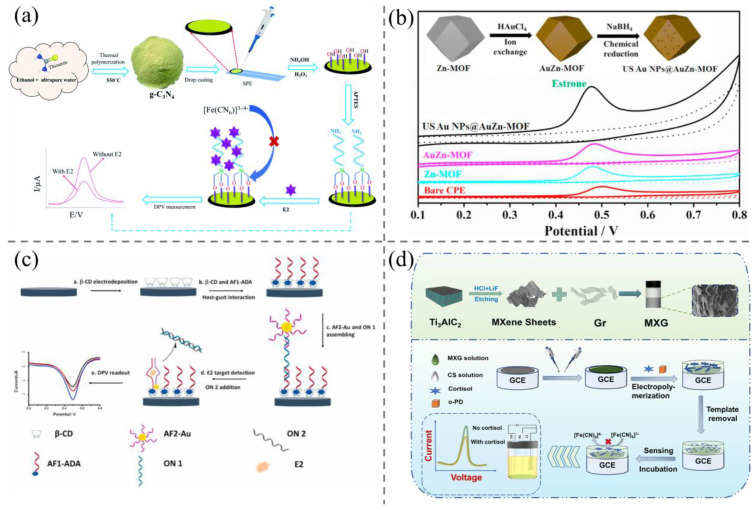
(**a**) The design principle and construction process of g-C_3_N_4_-based electrochemical biosensor for highly sensitive E2 detection [27]; (**b**) the fabrication of AuNPs@Zn–MOFs with enhanced electrochemical capacity, and their application in improving analytical performance of E2 [37]; (**c**) illustration of the aptamer recognition-based electrochemical sensor for E2 [28]; (**d**) illustration of the fabrication procedure and design principle of Ti_3_C_2_T_x_ MXene and graphene composite-based MIP electrochemical sensor for ultrasensitive cortisol detection [39].

The direct electrochemical detection of steroids was first introduced in 2009 [31]. However, the performance of conventional electrodes is compromised due to the formation of an oxidation product layer on the electrode surface, which hinders the detection of trace levels of steroid hormones using bare electrodes. To enhance sensitivity, a series of modifications have been implemented on the working electrode [32,33]. Precious metal nanoparticles, including those of copper, silver, palladium, and gold, are commonly utilized as electrode modification materials due to their favorable electrical conductivity, high specific surface area, and robust chemical stability [34]. Additionally, graphene and carbon nanotubes have been the subject of considerable research as potential modification materials for electrochemical sensors [35,36]. For instance, Bacchu et al. [27] developed an electrochemical sensor based on the modification of graphitic carbon nitride (g-C_3_N_4_) and 3-aminopropyltriethoxysilane (APTES) (Figure 2a). Initially, g-C_3_N_4_ was modified on screen-printed electrodes (SPE), resulting in a reduction of the electron transfer resistance (Rct) of g-C_3_N_4_/SPE compared to that of bare SPE. This observation suggests that g-C_3_N_4_ facilitates the creation of multiple active sites for electron transfer, thereby enhancing the conductivity of the modified electrode. Subsequent modification of APTES on the electrode entailed its adhesion to g-C_3_N_4_, a process that led to a reduction in the ΔEp value through enhanced electron transfer efficiency. Following the modification of APTES on the g-C_3_N_4_/SPE surface, a significant reduction in the Rct value of APTES/g-C_3_N_4_/SPE was observed. The large specific surface area and high electrocatalytic performance of graphitic carbon nitride can enhance the surface conductivity of the electrode and the recognition ability of the biosensor. Meanwhile, APTES has been shown to improve the stability and sensitivity of the electrochemical sensor, thereby optimizing the catalytic performance of the biosensor. The sensor was successfully employed for the detection of estradiol (E2) in milk, poultry meat, and environmental water samples, with a detection limit as low as 9.9 × 10^−19^ M.

Recently, metal–organic frameworks (MOFs) materials have also demonstrated promising potential for application in electrochemical analysis. MOFs are a novel class of crystalline nanoporous materials comprising metal nodes coordinated with organic ligands. These materials possess tunable pore structures and large specific surface areas, which affords them a range of potential applications. To further enhance the electrochemical sensing performance of MOFs, the effective strategy is to couple metal nanoparticles (MNPs) with MOFs. For example, Chai et al. [37] reported a gold nanoparticles (AuNPs)-embedded Zn–MOFs architecture based on in situ chemical reduction. The small-sized (5 nm) AuNPs could result in a notable increase in the density of active sites exposed by the gold oxides. The integrated, internal AuNPs markedly enhance the electron transfer capacity of MOFs, thereby providing a greater number of active catalytic sites. The MOFs inhibit the aggregation of AuNPs due to the support effect or the confinement effect, resulting in hybrids with enhanced electrochemical sensing performance (Figure 2b). The sensor demonstrated an exceptionally low detection limit for hormones, reaching as low as 12.3 nM. Furthermore, in terms of real sample assays, the spiked recoveries in real lake water samples exhibited a remarkable range from 93.0% to 103.5%, indicating excellent analytical performance.

The development of electrochemical aptasensors has attracted broad attention [38]. As representative examples of specific recognition element molecules, nucleic acid aptamers have attracted considerable attention in analytical and diagnostic applications in recent years. Nucleic acid aptamers are synthetic single-stranded nucleic acid sequences that exhibit high affinity and selectivity for target molecules through the formation of secondary structures. In comparison to traditional antibodies, nucleic acid aptamers possess distinctive advantages, including low cost, chemical stability, straightforward modification, favorable thermal stability, and straightforward in vitro synthesis, which permits more versatile design of diverse sensor types. Zhu et al. [28] developed an electrochemical sensor using an aptamer that was split into two fragments and labeled separately. In the presence of E2, the E2 aptamer undergoes reconfiguration on the electrode surface, resulting in the formation of a new configuration. The initial aptamer fragment is functionalized with adamantane and immobilized on the surface of the poly-β-CD-modified LSG electrode by combining it with the β-cyclodextrin (β-CD) cavities. The second aptamer fragment is modified on gold nanoparticles for use as an electrochemical indicator. The second fragment forms a secondary structure in the presence of E2, with the first fragment serving as a component. In the presence of E2, the two aptamer fragments underwent reorganization during binding, resulting in the AuNPs being positioned closer to the electrode surface. This led to a discernible alteration in the electrochemical signals (Figure 2c). The limit of detection was reduced to the femtomole level, with a detection limit of 0.7 pM.

In recent years, molecularly imprinted electrochemical sensors (MIECSs) have also been employed for the detection of steroids. Molecularly imprinted polymers (MIPs) are composed of polymer monomers that are polymerized with template molecules. Following the elution of the template molecules, a substantial number of holes remain on the surface of the polymer, which can exhibit high specificity for target molecules [40]. MIECSs can recognize the electrochemical signal changes that occur during the binding process between MIPs and target molecules. They are widely utilized in bioanalysis and other fields due to their low cost, high sensitivity and selectivity, short detection time, good environmental stability, and reusability [41]. They also have been adopted for steroid hormone detection [42]. For example, Liu et al. [39] developed an MIP biosensor by modifying MXG composites consisting of Ti_3_C_2_T_x_ MXene and graphene with a three-dimensional porous structure on a glassy carbon electrode (Figure 2d). When MXene is mixed with graphene (Gr), a 3D porous folded structure with a pore size of about 0.5–2 μm is formed. Gr and MXene have abundant surface groups and are adsorbed together through hydrogen bonding and van der Waals forces to form a stable porous structure that is neither agglomerated nor loose. The 3D porous structure facilitates rapid ion access to the electrode surface, allowing for electronic transitions and high electrical conductivity. At the same time, this structure provides a larger active surface area, which facilitates the adsorption of more substances. The CS/MXG/GCE–MIP sensor was then fabricated by electropolymerization using o-phenylenediamine monomer, cortisol template, and acetate. After removing the cortisol template, this biosensor was initially applied for specific electrochemical sensing of cortisol and subsequently used to detect cortisol in human saliva (Figure 2d). The MXG composites exhibit high electrical conductivity and a substantial electrically active surface area, which enhances the electrical conductivity of the biosensor. In this sensor, MXG porous composites were employed to modify the electrodes, resulting in an enhancement of the current response of the electrodes by a factor of more than 15, with a detection limit as low as 0.4 fM and a substantial linear range of 1 fM to 10 μM. Generally, to improve detection sensitivity and reduce assay time, rational choice of electrode and its surface modification with nanomaterials are necessary; however, they lead to complex designs and fabrication of MIECSs.

A field effect transistor (FET) is a type of transistor that uses an electric field to control the conductivity of charge carriers in a semiconductor material. This transistor has a high input impedance that attracts or repels charge carriers in and out. FETs and specific recognition elements (antibodies, nucleic acid aptamers, etc.) can be used to construct FET biosensors. After modifying the FET with a specific recognition element, the binding of the target molecule to the specific recognition element causes a change in the current near the FET [43], which is very weak and can be converted into a detectable electrical signal by the amplification effect of the FET on the current [44]. However, the current amplified by the FET is so weak that it needs to be measured by large precision devices, which limits the application of FET biosensors. To accommodate the need for in situ detection, researchers have developed silicon nanowire (SiNW)-based FETs, which are cumbersome and costly to prepare. Duan et al. [45] developed a novel p–n junction diode device architecture with vertically oriented SiNWs (vSiNWs), using an array of millions of n-doped vSiNWs, each of which is composed of the E2 receptor functionalized and acting as an anchor for the target hormone. This vSiNW diode biosensor can generate 100-fold higher current changes than conventional hSiNW-FET biosensors, significantly reducing instrument costs. Lee et al. [46] treated an immobilized antibody with a coupling of Horseradish Peroxidase (HRP) to the target molecule prior to detection, and then detected the target substrate in the sample by target displacement. The larger size of the protein and the charged groups on the surface increase the signal intensity and signal-to-noise ratio, and the pre-bound target organizes the binding of interferents to improve selectivity. The sensor was used for steroid concentrations in plasma, with an accuracy of up to 99%.

Electrochemical analysis stands as an efficacious means of detecting steroid hormones in food. Nevertheless, we acknowledge the persistence of several challenges that should be paid more attention, for instance, serious signal interference originating from complex food matrices, poor repeatability in the fabrication, and modification of metal electrodes. This requires researchers to further improve the sample pre-treatment methods and controllable surface functionalization of electrodes.

### 2.2. Colorimetric Methods

In colorimetric assays, the microscopic chemical changes of substances in solution can be reflected by visible color changes, which allows for the quantification of analytes by measuring changes in absorbance [47]. The field of analytical chemistry has witnessed a notable advancement in colorimetry in recent years, mainly due to the technique’s inherent simplicity of operation, high sensitivity, high accuracy, and low cost. The color change that can be observed without the aid of instrumentation facilitates high-throughput screening.

**Figure 3:**
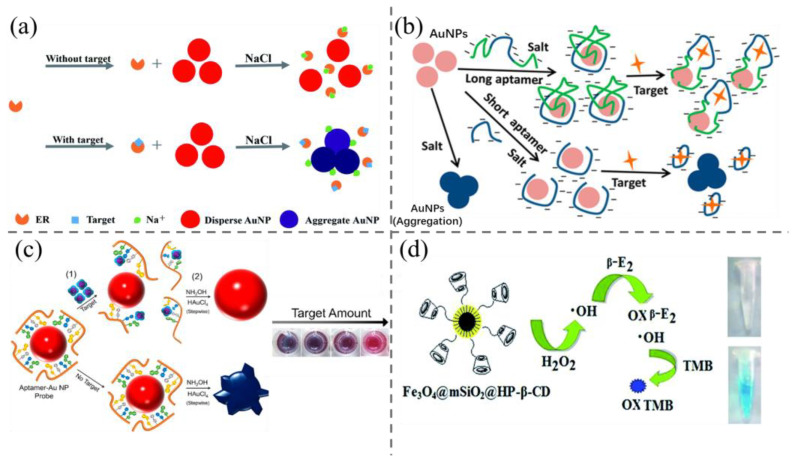
(**a**) The principle of Erα-induced AuNPs aggregation for E2 detection [52]; (**b**) aptamer length dominated AuNPs aggregation for visual detection of E2 [53]; (**c**) the design principle of colorimetric detection of small molecules in complex matrices via target-mediated growth of aptamer-functionalized AuNPs [54]; (**d**) principle diagram of a colorimetric assay for β-E2 based on the peroxidase-like activity of Fe_3_O_4_@mSiO_2_@HP-β-CD nanohybrids [55].

Among the numerous colorimetric methodologies, AuNPs have garnered significant attention. GNPs are sensitive colorimetric indicators due to their exceptionally high extinction coefficients and plasmonic excitation–absorption optical properties, which result in colors that are readily discernible to the unaided eye [48,49,50]. They have demonstrated great potential in quick and colorimetric detection of steroid hormone. For examples, Pule et al. [51] developed a colorimetric probe based on the incorporation of AuNPs into electrospun polystyrene nano-fibers and designed for in situ detection of E2. The probe is fabricated using an electrospinning technology, which allows for the synthesis of styrene–gold polymers in a simple and stable manner. As the concentration of estradiol increased, the surface plasmon resonance absorption peak shifted to the long wavelength band, and the visible color gradually changed from pink to blue. In the context of the detection of estradiol in dairy products, the visual detection limit was found to be 100 ng/mL. Liu et al. [52] employed a colorimetric sensor based on the non-targeted bio-recognitive “estrogen receptor alpha (ERα)” and AuNPs for the detection of multiple non-specific estrogens in tap water and milk samples (Figure 3a). ERα inhibits the aggregation of AuNPs in the absence of EDCs and recognizes multiple non-specific estrogens, which can be utilized for on-site high-throughput screening and provides a viable approach to initially screen for multiple EDCs simultaneously. The sensor has a detection limit of 0.01 nM; however, it is not specific for recognizing a single substance and is only applicable to high-volume screening, which has significant limitations.

Aptamer-linked AuNPs probes can enhance the specificity. However, the majority of long-sequence nucleic acid aptamers exhibit suboptimal sensitivity, which may be attributed to the formation of secondary structures that impair their sensitivity [18]. To improve the sensitivity, researchers have utilized a variety of strategies, including truncating or deleting segments of the aptamer sequence and regulating the extent of aptamer modification [18,53,54,56]. Liu et al. [18] reported that the adoption of the two shorter DNA sequences originating from the 76-mer E2 aptamer could enhance detection sensitivity. These results demonstrated that the split sequences retained the specificity and affinity of the original aptamer, while exhibiting a ten-fold increase in sensitivity, allowing for the detection of 0.1 ng/mL of E2. This improvement may be attributed to the shorter aptamer exhibiting a greater affinity for the AuNPs, which necessitates a lower concentration for adsorption. Alsager et al. [53] used new 35-mer and 22-mer aptamers to construct probes, resulting in a 25-fold increase in sensitivity in comparison with that obtained by the original 75-mer full-length aptamer. The authors of this study put forth a hypothesis that the inhibitory effect of the full-length aptamer on the target binding signal may be attributed to the presence of excess bases outside the binding region, which remain adsorbed on the AuNPs and prevent their aggregation (Figure 3b). In the same year, Soh et al. [54] developed a colorimetric sensor that employs aptamer-target recognition to facilitate the growth of aptamer-functionalized AuNPs. The morphology of AuNPs is contingent upon the concentration of the target and the quantity of aptamer modified on the surface of AuNPs, which will cause varying colors in solution (Figure 3c). Even minor discrepancies in the quantity of gold oxide result in discernible alterations in coloration. The sensitivity of this sensor for E2 is as low as 0.2 nM, which presents an additional potential avenue for enhancing the sensor’s sensitivity.

Additionally, colorimetric sensors based on nanozymes have attracted broad attention. For example, Fe_3_O_4_ has been demonstrated to possess peroxidase-like properties since 2007, and there is a substantial body of research investigating the potential use of Fe_3_O_4_ as an alternative to peroxidase. In comparison to horseradish peroxidase (HRP), Fe_3_O_4_ exhibits superior characteristics, including high stability at high pH, straightforward preparation and storage, and recyclability [55,57]. They will have great application potential in the colorimetric detection of steroid hormones. For example, Wei et al. [55] prepared Fe_3_O_4_@mSiO_2_ with magnetic and peroxidase-like activity, which was applied as a colorimetric sensor to detect E2 (Figure 3d). The sensor was then applied in meat food with a detection limit of 0.27 μM towards E2.

Presently, colorimetric-based assays continue to depend on external instrumentation. Furthermore, the pretreatment process necessitates the expertise of skilled operators. Additionally, the non-specific aggregation of probes can readily result in the occurrence of false positives. Consequently, the development of new materials based on more stable and simplified operation processes will be the future trend.

### 2.3. Fluorescence and Phosphorescence Method

**Figure 4:**
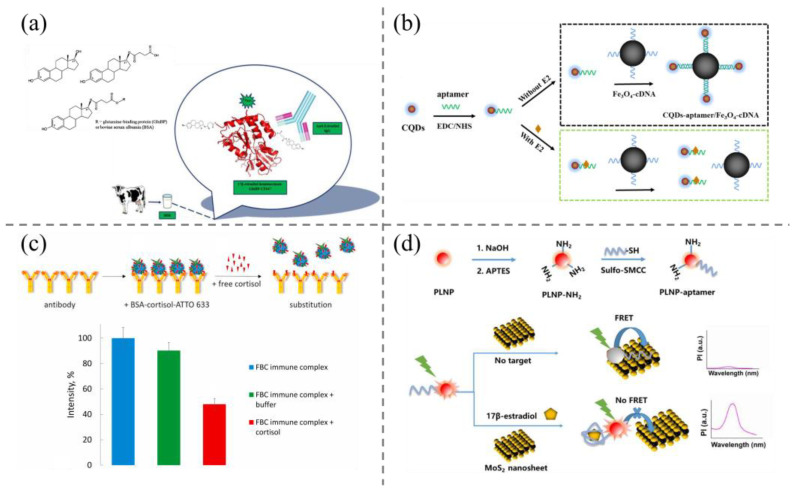
(**a**) Fluorescent probe Biotium CF647-based competitive immunoassay method for the determination of E2 in milk samples [61]; (**b**) schematic illustration of a FRET aptasensor for E2 detection based on CDs and Fe_3_O_4_ NPs [64]; (**c**) the design principle of metal-enhanced time-resolution fluorescence immunodetection of cortisol [72]; (**d**) schematic illustration of persistent luminescence resonance energy transfer (LRET) aptamer sensor for 17β-estradiol [73].

Fluorescence spectrometry is a frequently employed spectroscopic technique for the detection of steroids, due to its high sensitivity, high throughput, and minimal sample usage. The optical properties of fluorophores (e.g., quantum yield, excitation/emission wavelength, lifetime) are of paramount importance in fluorescence detection, as they directly determine the sensitivity of the target. The common fluorescent probes are organic dyes, quantum dots (QDs), carbon dots (QDs), long afterglow nanomaterials, and so on, of which fluorescence resonance energy transfer (FRET) is the dominant design principle for the detection of steroid hormones [58,59,60]. For example, Varriale and colleagues published a paper on the subject [61]. A synthetic estradiol-hemisuccinate-glutamine binding protein coupling was developed based on the fluorescent probe Biotium CF647 and a polyclonal monospecific anti-estradiol antibody for the determination of E2 in untreated milk samples via a competitive immunoassay (Figure 4a). The method does not necessitate any preliminary processing and exhibits a detection limit of 10 pM.

Semiconductor QDs featuring quantum size effects possess unique and superior optical characteristics, including narrow and symmetric excitation spectra and tunable emission spectra. In comparison to conventional organic fluorescent dyes, they exhibit superior characteristics, including a high fluorescence quantum yield and exceptional photostability [62], and thus QDs have revealed great advantages in steroids detection. For examples, Wang et al. [63] reported a QD-based FRET probe for megestrol acetate detection. In the FRET probe, water-soluble, low-toxicity β-cyclodextrin-functionalized ZnS quantum dots were first prepared. Cyclodextrin (CD) can serve as a molecular host, facilitating the formation of polymers with a range of guest molecules due to its distinctive molecular structure. Neutral red molecules can enter the water-soluble β-cyclodextrin cavity anchored on the surface of ZnS QDs in a neutral form and combine with the unique optical properties of the quantum dots to form FRET probes in a neutral form. The method was employed to ascertain the concentration of megestrol acetate in river water, with a detection limit of 0.0083 nM. Wei et al. [64] developed a novel fluorescent sensor by combining carbon dots (CDs) and magnetic Fe_3_O_4_ NPs (Figure 4b). This involved a partial modification of the competitive method: the carboxyl-rich CDs were labeled with an E2 aptamer, and the complementary DNA sequences were modified in the same way on Fe_3_O_4_. This prevented the aptamer from hybridizing with its complementary DNA. When separated using a magnet, the fluorescence intensity of the supernatant is markedly reduced in the absence of E2. Conversely, the presence of E2 prevents the formation of the complex, resulting in an elevated fluorescence intensity of the supernatant. The concentration of E2 can be quantified based on the degree of increase in fluorescence intensity. The sensor demonstrated a detection limit of 3.48 pM and finally was successfully applied for accurate E2 detection in milk and water samples. In addition, several other fluorescent sensors have been developed based on the FRET principle employing alternative fluorophores, such as organic dyes [65,66,67], cyclodextrins [68,69], and up-conversion nanoparticles (UCNPs) [70,71], as the energy donors.

In recent years, there has also been significant advancement in the development of fluorescent sensors with metal-enhanced fluorescence strategies. Safarian et al. [72] described a metal-enhanced fluorescence assay based on a displacement of a dye-labeled BSA-cortisol conjugate from the immune complex immobilized on the golden islands by free cortisol (Figure 4c). This competitive approach allows time-resolved monitoring of the fluorescent signal, surface-enhanced by the gold film, and provides the possibility of continuous real-time cortisol monitoring based on the implantable surface-enhanced im-munosensor. The target molecule can be identified through time-resolved detection by measuring the fluorescence intensity following the addition of the sample, with a detection limit of 20 ng/mL.

As a spectroscopic technology, fluorescence assays inevitably involve serious autofluorescence and scattered light under the excitation of ultraviolet/blue light, which cause decreased signal-to-noise ratios or sensitivity [74]. Long lifetime-based delay detection technology is an ideal solution. Persistent luminescence nanoparticles (PLNPs) can remain emissive and detectable after removing the excitation, and thus effectively eliminate the above optical interference and markedly enhance the detection signal-to-noise ratio [75,76]. Based on this consideration, Zhang et al. [73] reported a FRET probe for E2 detection using PLNPs and MoS_2_ nanosheets as the energy donor and the energy acceptor, respectively (Figure 4d). The molecular recognition unit, i.e., E2 aptamer, was covalently linked on the surface of PLNPs. The FRET sensor revealed a limit of detection of 0.29 ng/mL, and was successfully applied for the detection of E2 in complex milk samples, demonstrating its good capacity in the removal of optical interference.

Despite luminescence-based technologies having achieved great advancement in steroid hormones detection, there are several insufficiencies to be addressed, such as the use of spectrophotometers, time-consuming pre-treatment steps, and the optical interference (e.g., autofluorescence and scattered light) originating from the excitation light and the complex food matrix. Thus, the development of more convenient and reliable matrix purification methods, as well as portable fluorescence detection equipment with anti-interference capability for on-site detection, is the trend of the future.

### 2.4. Surface-Enhanced Raman Spectroscopy Detection Technology

Raman spectroscopy is a significant analytical method for identifying and characterizing the chemical structure of molecules, analogous to infrared spectroscopy, which is an ideal technology for analyzing molecular vibrational spectra. Raman spectroscopy offers several advantages, including rapid analysis, straightforward methodology, and suitability for on-site testing [20,77]. The detection and analysis of scattered light enables the acquisition of information pertaining to the vibrational state of the sample molecules. The stretching vibration of O-H, the bending vibrations of C-H and O-H, and the ring deformation in the C18 backbone structure of steroids result in characteristic peaks in the Raman spectra of steroid hormones, which can be identified. Nevertheless, the relatively low intensity of the Raman scattering effect and the relatively low steroid content in actual samples restrict the applicability of this method for the detection of steroids [78,79]. To obtain more robust Raman scattering signals and enhance the signal-to-noise ratio, researchers have devised surface-enhanced Raman spectroscopy (SERS) detection techniques to achieve highly sensitive detection of target molecules.

SERS substrates are of paramount importance in the design of SERS probes and can be classified into two principal categories: colloidal and solid substrates. The most commonly-adopted colloidal substrates are gold, silver, and gold–silver core–shell nanoparticle colloids [80,81,82]. However, colloidal suspensions are inherently unstable and tend to agglomerate. While solid substrates can address this limitation, they are more challenging to fabricate. The amplification of Raman signals is typically regarded as a synergistic phenomenon, whereby electromagnetic and chemical enhancements interact to produce a greater effect than would be expected from either mechanism alone. The excitation of light by precious metal nanoparticles, such as gold and silver, with high transition frequencies and high dielectric constants, produces localized surface plasmon resonances, which in turn lead to electromagnetic enhancement [83]. Among the various noble metal nanoparticles, silver nanoparticles (AgNPs) exhibit the most pronounced SERS activity and are more cost-effective than AuNPs, rendering them optimal SERS substrates [84].

**Figure 5:**
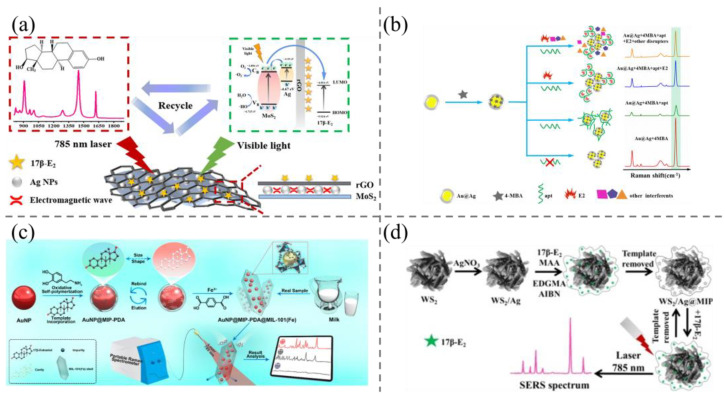
(**a**) Schematic illustration of MoS_2_/Ag@rGO-based SERS detection and photocatalytic degradation of 17β-E2 [83]; (**b**) illustration of Au@Ag substrate-based SERS aptasensor for E2 using 4-MBA as Raman reporter molecule [85]; (**c**) illustration of the design and fabrication of molecularly-imprinted AuNPs-embedded substrate Fe–MOFs for highly selective SERS detection of 17β-estradiol in milk [86]; (**d**) the fabrication of sandwich WS_2_/Ag@MIP composite substrate for ultrasensitive SERS detection of 17β-estradiol in food [87].

Recently, Liu et al. [83] selected AgNPs and self-cleaning MoS_2_ as the SRES source and the substrate, respectively. In addition, reduced graphene oxide (rGO) was wrapped around the AgNPs to serve a protective role and adsorb the target (Figure 5a). This configuration reduced the distance between the target molecules and the AgNPs, thereby enhancing the SERS signals. MoS2 has a large specific surface area, which provides a platform for the regular arrangement of AgNPs and higher sensitivity. This SERS probe could detect E2 as low as 5 pM and was successfully applied for E2 detection in farm wastewater samples.

Molecularly imprinted polymers and aptamers were introduced into SERS probes to improve specificity in steroid hormone sensing [87,88,89]. For examples, Liu et al. [85] developed a novel SERS system using gold–silica core–shell nanoparticles (Au@Ag CS NPs) as a substrate combined with aptamers. 4-Mercaptobenzoic acid (4-MBA) was utilized as a Raman reporter molecule for the detection of E2 in complex water samples. Due to the electrostatic interactions between the aptamer and the Au@Ag CS NPs, Au@Ag CS NPs, and 4-MBA were separated, resulting in a reduction in Raman signal intensity (Figure 5b). Upon the addition of E2 to the system, it was observed that the aptamer was preferentially bound, forming a complex that resulted in the separation of the nanoparticles from the aptamer. The Raman scattering intensity of the reporter molecule was then recovered, thus allowing for the quantification of E2 by monitoring the change in Raman scattering intensity before and after the addition of E2. The sensor is sufficiently sensitive to detect trace E2 in water samples that cannot be detected by HPLC, with a detection limit as low as 0.05 pM, a linear range of 0.1 pM–10 nM, and good selectivity towards E2. Zhang et al. [86] embedded polydopamine (PDA) molecularly imprinted gold nanoparticles with a core–shell structure into a ferrometallic organic framework material to form a hexagonal microspindle structure (Figure 5c). The AuNP@MIP-PDA was encapsulated in MIL-101 crystals through the hydrophobic interaction between the organic ligands and the aromatic groups of PDA, the chelation of the catechol groups, and the introduction of acetic acid. Combining the SERS activity of AuNPs, the specific recognition sites of MIPs, and the adsorption and enrichment ability of MIL-101, the nanohybridization was highly selective. The sensor can be directly applied to the detection of E2 in milk without pre-processing, with a detection limit of 0.195 fM. More recently, Liu et al. [87] constructed a sensitive and recyclable SERS substrate for the detection of E2 in chicken meat and milk, with AgNPs as the SERS source and MIP as the shell, which selectively enriches the target molecules, distances the target molecules from the SERS source, and protects the AgNPs, and the detection limit was as low as 0.0958 pM (Figure 5d).

SERS has demonstrated considerable potential as an ultrasensitive detection technique for the determination of steroid hormone residues in food. SERS boasts high sensitivity and minimal pretreatment requirements. However, complex interferences present within the food matrix can bring serious signal interference and lead to poor specificity. In addition, the stability and reproducibility of SERS substrate should be enhanced in subsequent developments.

### 2.5. Lateral Flow Immunochromatography

**Figure 6:**
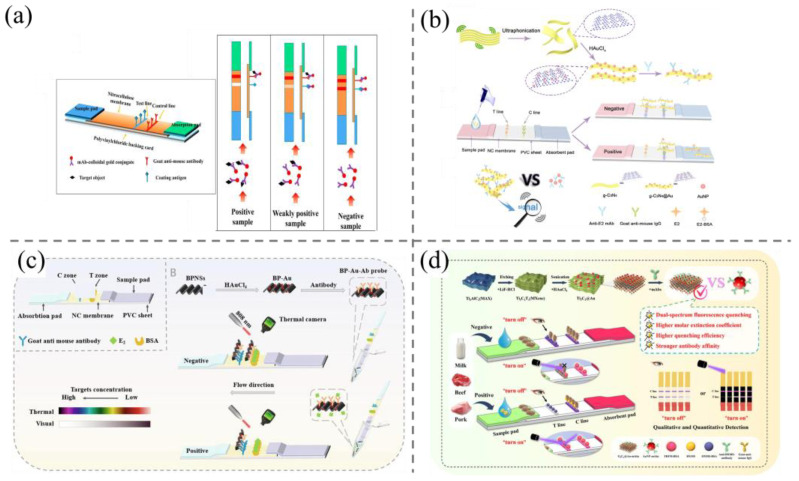
(**a**) Colloidal AuNPs-based LFIA for the rapid detection of three natural estrogens in milk [93]; (**b**) illustration of the fabrication of g-C_3_N_4_ nanosheets–Au composites nanotag and its application in LFIA for E2 detection [94]; (**c**) schematic illustration of the construction of BP–Au-based colorimetric and photothermal dual channel LFIA test strips for E2 detection [102]; (**d**) schematic illustration of the construction of Ti_3_C_2_@Au-based colorimetric-fluorescence probe for the LFIA of dexamethasone [105].

Lateral flow immunochromatography assays (LFIAs) are employed extensively in clinical diagnostics, environmental monitoring, and food safety due to their sensitivity, low cost, ease of operation, and suitability for rapid on-site detection [90,91]. The most commonly utilized tags in LFIA techniques is colloidal AuNPs [92]. In recent years, colloidal AuNPs-based LFIAs have been exploited for the quick detection of steroid hormones in foodstuffs. For examples, Wang et al. [93] reported an AuNPs-based LFIA test strip for multiple screening of estrone, estradiol, and estriol in milk samples, with visual detection limits down to 5 ng/mL (Figure 6a). To enhance sensitivity, Yao et al. [94] developed a g-C_3_N_4_ nanosheets–Au composites nanotag in the design of highly sensitive LFIAs. The g-C_3_N_4_@Au composites display a darker color and higher absorption capacity than AuNPs, which effectively enhances the signal intensity (Figure 6b). As expected, the fabricated test strips revealed an enhanced visual detection limit of 0.5 ng/mL. This method was then applied for the E2 residual quick screening in four kinds of food samples including fish, shrimp, pork, and chicken.

Despite LFIAs being extremely versatile, they are still restricted to the qualitative detection of a highly concentrated analyte and incapable of working for low-concentration analyte detection, especially in complex food matrices, due to serious background signals [95,96,97,98]. Numerous studies have confirmed that the detection sensitivity is closely associated with the label probes used [99,100,101]. Diverse label probes like fluorescence microspheres, colored latex microspheres, Prussian blue nanoparticles, and graphene oxide were adopted for the design of steroid hormone LFIAs [102,103,104]. Recently, a photothermal lateral flow immunochromatographic strip was developed for E2 detection (Figure 6c) using black phosphorus (BP)–Au nanocomposites as the label probes [102]. The signal acquisition of photothermal LFIAs relies solely on a light source and a portable infrared thermal imager, which can reduce the cost of testing. Anti-estradiol-modified BP–Au nanocomposites were employed as photothermal contrast signal probes, with the temperature of the test line recorded by an infrared camera. Under the optimal detection conditions, the test strip demonstrates a competitive detection limit of 50 pg/mL, thereby enabling the rapid screening analysis of E2 in water, milk, and milk powder samples.

In recent years, LFIA test strips with dual-signal output have been paid more attention due to their merits of self-correcting and good reliability [106,107,108,109]. To illustrate, Deng et al. [105] developed dual-signal LFIA test strips using Ti_3_C_2_@Au fluorescent-colorimetric probes, which were utilized in both the “off” and “on” modes. Ti_3_C_2_@Au nanocomposites exhibit high molar extinction coefficients, quenching ability, high dispersibility, and biocompatibility, which enable high sensitivity at low antibody levels. The synthesis process does not require any organic reagents or heating reactions, allowing for the detection of low antibody levels with high sensitivity. The time-resolved fluorescent microspheres and target molecules are immobilized in the T-line. In the presence of a negative charge, the probe is captured by the T-line, resulting in the quenching of the microspheres’ fluorescence and the appearance of a colorimetric signal. Conversely, in the presence of a positive charge, the probe is unable to bind to the T-line, leading to the restoration of the microspheres’ fluorescence and the dissolution of the probe aggregates within the T-line, thus eliminating the colorimetric signal (Figure 6d). By combining the “off” and “on” modes, the two signals are calibrated against each other, thereby enhancing the reliability of the signal. This method was employed for the determination of dexamethasone in milk, beef, and pork, with detection limits of 0.0013, 0.080, and 0.070 μg/kg, respectively.

In summary, LFIAs are competitive in the quick detection of steroid hormones and have achieved positive progress in recent years. However, label probes with high signal-to-noise ratios should be introduced to enhance sensitivity and reduce false negatives. In addition, the development of high-affinity monoclonal antibodies is essential in the construction of LFIAs for specific steroid hormone detection.

### 2.6. Surface Plasmon Resonance Method

Surface plasmon resonance (SPR) is an optoelectronic phenomenon in which incident light, incident at a specific angle on a plasma metal surface (e.g., gold, silver), excites surface-bound electromagnetic waves in the ionized metal. The binding of biomolecules will result in localized changes in the refractive index of the ambient medium, which can be detected. By monitoring the change in the intensity polarity of the reflected light over time, the reaction process is monitored and converted into a readable signal, allowing quantification of the target concentration [110,111]. SPR possesses the following properties: it is label-free, real time, sensitive, and quantitative [112,113]. SPR biosensors have been utilized for the detection of large molecules, such as proteins. However, for samples with concentrations less than 1 pmol/L and molecular weights less than 8000 Da, the changes in refractive indices induced by the compounds are so small that SPR cannot detect them [114,115].

**Figure 7:**
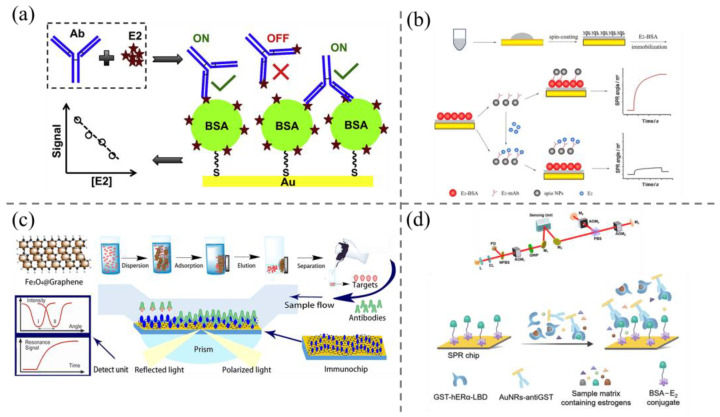
(**a**) Schematic diagram of SPR biosensor competitive method for E2 determination [116]; (**b**) schematic diagram of magnetic nanoparticle-enhanced SPR sensor for E2 detection [118]; (**c**) illustration of the design of graphene-based amplified signal SPR sensor for the detection of three estrogens in milk [119]; (**d**) schematic illustration of the fabrication principle of SPR chip and the detection mechanism of estradiol detection [120].

In order to enhance the sensitivity of SPR biosensors for detecting small molecule target substances, researchers have explored various methodologies. Among these approaches is the enhancement of the utilization of specific recognition substances. The antigen is labeled with a high molecular weight antibody and detected competitively with the unlabeled sample antigen. Alternatively, small molecule antigens are coupled to the surface of the sensor, a primary antibody is mixed with the free antigen-containing sample for competitive detection, and the signal is further enhanced by the secondary antibody [114]. For Cao et al. [116], a detection limit of 0.3 pmol/L was obtained by immobilizing the complete antigen on an SPR chip, thereby allowing free E2 to compete with the complete antigen on the surface of the SPR chip for the binding site of the antibody and detecting estrone (E1) by SPR (Figure 7a). The use of MIP as a biorecognition material to improve the sensitivity of SPR sensors has also attracted the attention of researchers in recent years. In the study by Blackburn et al. [117], MIP compounds targeting four glucocorticoids, including dexamethasone, were synthesized by solid-phase synthesis. These compounds were then utilized for the detection of the aforementioned targets in simulated agricultural waste samples. The detection limits of these compounds ranged from 1.3 to 6.5 nM, while their linear ranges extended from 15.9 to 62.8 nM. The findings indicated that the MIP compounds exhibited suitability for the determination of targets in simulated agricultural waste samples.

The sensitivity of SPR sensors can be enhanced by nanomaterials, especially noble metal nanoparticles. In recent years, nanoparticles such as AuNPs, AgNPs, SiO_2_NPs, PdNPs, and PtNPs have been extensively utilized as amplification modulators. The SPR signal can be enhanced by metal nanoparticles, and the use of additional labeling is not required. This finding was reported by Moses et al. [121], where a novel, label-free sensing method for the detection of trenbolone was developed. This method utilizes AgNPs as amplification modulators, with trenbolone acetate/silver nanoparticles (Tren Ac/AgNPs) complexes playing a key role. Through meticulous calculation of molecular behaviors, it was determined that O^2−^ on trenbolone can interact with Ag to form a complex affecting plasmonic excitations. Notably, the detection limit of this method is reported to be as low as 9.12 ppb, a significant advancement in the field.

The integration of the benefits inherent to specific recognition of substances and nanoparticles has the potential to further enhance the performance of SPR sensors. Jia et al. [118] undertook the development of SPR biosensors incorporating magnetic nanoparticles (MNPs) as signal amplification elements, thereby enhancing SPR signaling through the coupling of E2 antibodies to MNPs. This approach yielded a detection limit of 0.81 ng/mL, with a linear range extending from 1.95 to 2000 ng/mL, as determined through a competitive method for the detection of E2 in milk samples (Figure 7b) [119]. The detection of E2, DES, and BPA in milk was achieved by an indirect competitive method through magnetic dispersive solid-phase extraction (MDSPE) with graphene and carboxylated graphene oxide (GO-COOH) material as an amplification component, which was modified on an Au chip to immobilize the complete antigens (Figure 7c). The detection limits of the sensor in milk samples were as low as 2.901 × 10^−6^–5.169 × 10^−5^ ng/mL.

Concurrently, methodologies for enhancing optical structures and detection systems to augment sensitivity are undergoing continual development. As evidenced by the research of Tan et al. [115], a polarized light-compensated SPR system with laser heterodyne feedback was developed to significantly amplify refractive index (RI) intensity changes caused by molecule/biomolecule binding when the SPR is excited at the surface using laser heterodyne feedback interferometry (LHFI). The effects of mechanical, thermal, and laser noise can be minimized by a common path compensation method that combines orthogonally polarized light with frequency multiplexing (OLFM). Furthermore, the SPR signal was enhanced by optimizing and designing gold nanorods (AuNRs) through finite-difference time-domain (FDTD) simulation (Figure 7d). When detecting E2 with an E2 receptor as the recognizing substance, a detection limit of 0.004 ng/L was obtained, which was 180-fold lower than that of the system without the introduction of AuNRs.

Currently, the SPR method is still less used in the detection of food samples. The SPR assay boasts several advantages, including sensitivity, rapidity, and the capability for real-time monitoring. However, it is imperative to note that the SPR assay technique is highly susceptible to variations in sample composition and temperature, which can potentially compromise the accuracy of the results. Furthermore, the acquisition and maintenance of SPR detection equipment can be costly, necessitating the expertise and experience of operators. Improper operation of this equipment can also adversely affect the accuracy of the results. The cost-effectiveness of equipment and the optimization of detection processes may emerge as a focal point for future research endeavors.

## 3. Conclusions and Future Perspectives

In summary, a comprehensive review on recent advancements in rapid detection techniques for steroid hormones was presented, encompassing electrochemical methods, colorimetric assays, fluorescence techniques, SERS spectroscopy, and LFIAs. We have summarized the advantages and disadvantages of the various rapid testing methods in Table 1. In addition, a comparison of current rapid testing methods for steroid hormones is shown in Table 2. Furthermore, the research progress of these methodologies in detecting steroid hormones within food matrices, specifically animal-derived foods like dairy products and meats, over the past decade was examined. In contemporary times, hormones, particularly endocrine-disrupting chemicals, pose a widespread and persistent threat to human health and wellbeing. These substances are ubiquitous in the global environment and possess the potential to adversely impact human health. In recent years, numerous food safety incidents have occurred across various countries worldwide. Given the swift market turnover and trade demands, there is an urgent necessity for the development of novel detection methods. These methods must be swift and highly sensitive, capable of instantaneously detecting trace substances. This will require the selection and design of ultra-sensitive signaling probes, along with the recognition of molecules, encompassing the preparation of highly specific antibodies, screening of functional ligands, and more. It is also plausible that the future will see a simplification of data processing, with the use of artificial intelligence (AI) becoming a prevalent trend. One such application would involve the automatic analysis and processing of captured images, with the subsequent calculation of output results following signal capture via LFIA. This would result in a significant reduction in inspection time and an enhancement in inspection efficiency. In addition, the utilization of AI will provide an opportunity in multi-channel signal processing and big data acquisition and would provide a better model in the prediction and control of steroid hormones food contamination.

Currently, the detection of steroid hormones primarily focuses on estradiol due to its status as the most potent steroid hormone. However, the detection of other steroid hormones, such as androgens like methyltestosterone and testosterone propionate, as well as glucocorticoids such as dexamethasone and betamethasone, is scarcely reported in scientific literature. This underscores the critical need for the integration of high-throughput detection technologies, such as microfluidics, array sensing, and similar techniques, to achieve the simultaneous identification and detection of multiple steroid hormones. In addition, the development of sensitive, portable, low-cost detection devices and miniaturized instruments faces great challenges, yet is also meaningful. Moreover, in situ detection technology of endocrine-disrupting chemicals, including steroids, remains an unresolved challenge. In situ analysis and real-time monitoring are also pivotal for controlling the entry of these substances into the food chain, which may emerge as a research priority in the future.

## Figures and Tables

**Table 1:** Advantages and disadvantages of varying methods for quick detection of steroid hormones.

**M** **ethodologies**	**Advantages**	**Disadvantages**
Electrochemical method	Fast response, low cost, and high sensitivity	Relatively low stability, complex electrode modification, and poor reproducibility
Colorimetric method	Visualization, simple, and ready for on-site analysis	Low sensitivity and poor selectivity (non-specific aggregation of probe causes false positive)
Fluorescence method	High sensitivity and high-throughput	Serious optical interference, complicated biomodification
SERS	Rapid, sensitive, non-destructive, in situ analysis	Complicated design and fabrication of SERS substrate, relatively low stability, and poor repeatability
LFIAs	Good specificity, relatively high sensitivity, low cost, easy to operate, rapid and on-site detection	Poor reproducibility, instability of the antibody, difficult to quantify
SPR	Sensitive, fast, real-time monitoring	Sensitive to substrate and temperature, high equipment cost, requires specialized personnel to operate

**Table 2:** Application of different detection methods for steroid hormones detection in food matrices.

Methodologies	Samples	Targets	Detection Limit	Linear Range	Recovery	Analysis Time	Refs.
Electrochemical method	Milk	Bisphenol A	5 nM	0.01–10 μM	90–116%	30 min	[122]
	Milk, Meat	Estradiol	9.9 × 10^−19^ M	10^−18^–10^−6^ M	95.1–104.8%	20 min	[27]
Colorimetric method	Milk	Estradiol	13.1 pM	0.05–0.8 nM	100.1–113.0%	1.5 h	[56]
		Bisphenol A	7.60 pM	10–100 pM	96.10–106.5%	55 min	[123]
	Meats	Estradiol	0.15 μM	0–10 μM	95.70–113.8%	40 min	[57]
Fluorescence method	Milk	Progesterone	0.065 ng/mL	0.25–25 ng/mL	90–105%	15 min	[124]
		Estradiol	3.48 × 10^−12^ M	10^−11^–10^−6^ M	82–107%	1.5 h	[125]
			0.29 ng/mL	0.5 ng/mL–1.2 μg/mL	93.6–102.4%	30 min	[73]
SERS	Chicken, Milk	Estradiol	9.58 × 10^−14^ M	10^−12^–10^−4^ M	95.00–98.72%	1 h	[87]
	Milk		1.95 × 10^−16^ M	10^−14^–10^−6^ M	90.56–109.40%	35 min	[86]
LFIAs	Aquatic products, milk	Estradiol	65 ng/g	75 ng/g	-	7–10 min	[92]
	Chicken, Pork	Testosterone Propionate	0.32 μg/kg	0.05–0.94 ng/mL	90–110%	40 min	[126]
	Milk	Dexamethasone	0.0013 μg/kg	0.05–0.08 μg/kg	81.1–113.7%	20 min	[105]
	Beef		0.08 μg/kg	0.35–1.50 μg/kg	89.2–115.4%		
	Pork		0.07 μg/kg	0.25–1.00 μg/kg			
SPR	Water	Estradiol	2.50 × 10^−13^ mol/L	2.50 × 10^−13^–2.50 × 10^−9^ mol/L	96.1–101.4%	10 min	[120]
	Milk	Estradiol	0.81 ng/mL	1.95–2000 ng/mL	99.63–114.91%	30 min	[118]
	Milk	Estradiol	3.605 × 10^−6^ ng/mL	2.105 × 10^−5^–1.403 × 10^−2^ ng/mL	87.26–105.30%	20 min	[119]
		Diethylstilboestrol	2.901 × 10^−6^ ng/mL	1.687 × 10^−5^–1.141 × 10^−2^ ng/mL			
		Bisphenol A	5.169 × 10^−5^ ng/mL	2.288 × 10^−4^–9.211 × 10^−2^ ng/mL			
